# Identification of the Shared Gene Signatures of HCK, NOG, RNF125 and Biological Mechanism in Pediatric Acute Lymphoblastic Leukaemia and Pediatric Sepsis

**DOI:** 10.1007/s12033-023-00979-6

**Published:** 2023-12-20

**Authors:** Ying-Ping Xiao, Yu-Cai Cheng, Chun Chen, Hong-Man Xue, Mo Yang, Chao Lin

**Affiliations:** 1 Pediatric Hematology Laboratory, Division of Hematology/Oncology, Department of Pediatrics, The Seventh Affiliated Hospital, Sun Yat-sen University, Shenzhen, 518107 Guangdong China; 2 Scientific Research Center, The Seventh Affiliated Hospital, Sun Yat-sen University, Shenzhen, 518107 Guangdong China

**Keywords:** Pediatric acute lymphoblastic leukaemia, Pediatric sepsis, Shared genes, Biological mechanism, Diagnosis

## Abstract

The shared mechanisms between pediatric acute lymphoblastic leukaemia (ALL) and pediatric sepsis are currently unclear. This study was aimed to explore the shared key genes of pediatric ALL and pediatric sepsis. The datasets involved were downloaded from the Gene Expression Omnibus (GEO) database. Differentially expressed genes (DEGs) between disease and control samples in GSE13904 and GSE79533 were intersected. The least absolute shrinkage and selection operator (LASSO) and the boruta analyses were performed in GSE13904 and GSE79533 separately based on shared DEGs, and shared key genes were obtained by taking the intersection of sepsis-related key genes and ALL-related key genes. Three shared key genes (*HCK*, *NOG*, *RNF125*) were obtained, that have a good diagnostic value for both sepsis and ALL. The correlation between shared key genes and differentially expressed immune cells was higher in GSE13904 and conversely, the correlation of which was lower in GSE79533. Suggesting that the sharing key genes had a different impact on the immune environment in pediatric ALL and pediatric sepsis. We make the case that this study provides a new perspective to study the relationship between pediatric ALL and pediatric sepsis.

## Introduction

Pediatric acute lymphocytic leukemia (ALL) is a representative hematological malignancy among children, and the incidence reaches 3/100,000–4/100,000,000, taking up approximately 75–80% of childhood acute leukemia [1, 2]. In the children ALL diagnosis recommendations (fourth revision), the original + naive lymphocyte ratio in ALL children exceeds 25% in the diagnosis of ALL. The main clinical manifestations were infection, bleeding, anemia and extramedullary tissue and organ infiltration, the accurate cell morphology (morphology, M) -immunology (immunology, I) -genetics (cytogenetics, C) -molecular biology (molecular biology, M) classification contributes to the ALL’ clinical classification and the establishment of effective treatment [3, 4]. Despite advances in treatment, this type of cancer remains the leading cause of death among children worldwide. Identifying new biomarkers has important scientific implications for understanding the complex molecular mechanisms of the ALL [5]. Sepsis is a kind of organ dysfunction disease that threatens patients’ life, which is mainly resulted from the dysregulated response to infection by host [6]. There are about 1.2 million newly diagnosed cases around the world each year [7]. As reported, children hospitalized with sepsis present a mortality rate of 25–50% [8]. Despite the several times of revision about the definition of sepsis, the latest revised definition of adult sepsis cannot be applied to children strictly, hence it is critical to conduct early diagnosis and effective management so as to improve the clinical outcomes of children with sepsis risk [9]. Therefore, understanding the underlying molecular mechanisms of sepsis is essential for identifying early diagnostic biomarkers and finding effective drugs [10].

The chemotherapy process of ALL children is accompanied by infection caused by various pathogens, such as bacteria and fungi. Infection is capable of prolonging the duration of chemotherapy as well as negatively impacting the chemotherapy effect, and serious infection can lead to death [11]. As ALL itself is accompanied by lower body immunity and drugs used for chemotherapy can cause marrow suppression, and neutrophil deficiency, infection risk can be increased in children. According to previous literature studies, 2–3% of ALL children die because of infection, thereinto, sepsis is the mainstream death reason [12]. In addition, the presence of premature sepsis significantly affects the prognosis of ALL. To improve the outcome of ALL treatment, interventions targeting sepsis may lead to better outcomes for patients [13, 14].

Bioinformatics analysis that applies computational biology in medicine treatment has been a hot research area in biomedical research presently. Sepsis and ALL are both malignant diseases that seriously threaten the health and life of children. Identifying differential expressed genes (DEGs) and exploring their molecular mechanisms in Sepsis and ALL can provide potential targets for disease prevention and treatment [15, 16]. Considering the clinical correlation between ALL and sepsis mentioned above, but the lack of common research targets for both, this study for the first time combined the gene expression profiles of both to screen biomarkers, explore their common genes, biological functions and molecular mechanisms, and provide theoretical basis for exploring new target drugs.

## Materials and Methods

### Data Source

Two pediatric sepsis datasets (GSE13904 and GSE26440) and two pediatric ALL datasets (GSE79533 and GSE48558) were downloaded from the GEO database (https://www.ncbi.nlm.nih.gov/gds). Eighteen normal control blood samples and 52 septic blood samples from the GSE13904 [17] dataset were included. Thirty-two normal control blood samples and 98 sepsis blood samples from the GSE26440 [18] dataset were included. Three normal control blood B-cell samples and 226 ALL blood B-cell samples from the GSE79533 [19] dataset were included. Eleven normal control blood B-cell samples and 27 ALL blood B-cell samples from the GSE48558 [20] dataset were included (Table 1).The source code and data for this study are available on the website (https://doi.org/10.6084/m9.figshare.24460009).

**Table 1:** Information of datasets

	Dataset source	Sample source	Control sample	Disease sample	References
GSE13904	Pediatric sepsis datasets	Whole blood samples from children	18	52	PMID: 19325468
GSE26440	Pediatric sepsis datasets	Whole blood samples from children	32	98	PMID: 24650276
GSE79533	Pediatric ALL datasets	Blood B-cell samples	3	226	PMID: 27634205
GSE48558	ALL datasets	Blood B-cell samples	11	27	PMID: 23836560

GSE79533 is pediatric ALL datasets, and GSE48558 is ALL datasets, but it is uncertain whether it is a sample for children. The samples of both datasets are derived from blood B-cell samples. The dataset for acute lymphoblastic leukemia in children is limited, and the dataset with human B-acute lymphoblastic leukemia cells (B-ALL) is selected as external validation here

### Identification of Shared DEGs

Differential expression analysis was performed to screen DEGs between disease samples and control samples using the “limma” package [21] in GSE13904 and GSE79533 separately, by setting |logFC|> 0.5 and adj.P.Val < 0.05. The intersection of the DEGs obtained from GSE13904 and GSE79533 was defined as the shared DEGs. To explore the potential mechanisms of shared DEGs, Gene Ontology (GO) and Kyoto Encyclopedia of Genes and Genomes (KEGG) enrichment analysis were completed using the clusterProfiler package [22].

### Protein–Protein Interactions (PPI) Network Analysis

To explore whether interactions existed among the shared DEGs, a PPI network was created using STRING database (https://cn.string-db.org/). Expression box plots of shared key genes in the four datasets were plotted using ggplot2.

### Identification of Shared Key Genes

The Least absolute shrinkage and selection operator (LASSO) analysis was performed in GSE13904 and GSE79533 separately based on shared DEGs with the glmnet package. Similarly, the boruta analysis was performed in GSE13904 and GSE79533 separately based on shared DEGs with the Boruta package [23]. The sepsis-related key genes and ALL-related key genes were obtained by taking the intersection of signature genes from LASSO and boruta analysis. Shared key genes were obtained by taking the intersection of sepsis-related key genes and ALL-related key genes.

### Evaluation of the Diagnostic Performance of Shared Key Genes

To further confirm the ability of shared key genes to distinguish disease samples from normal control samples, Receiver Operating Characteristic (ROC) curves for shared key genes were plotted in each of the four datasets included in this study. ROC curves were plotted by pROC package [24].

### Immune Infiltration Analysis and Drugs Prediction

The immune abundance of 22 immune cells in disease and normal control samples of the GSE13904 and GSE79533 were calculated using the CIBERSORT algorithm [25] to find differentially expressed immune cells. Correlations were also calculated between shared key genes and 22 immune cells in the GSE13904 and GSE79533 datasets, respectively. Potential target drugs for shared key genes were predicted using the Comparative Toxicogenomics Database (CTD) (http://www.ctdbase.com/) and visualized using Cystocape.

### Statistical Analysis

The statistical analyses in this study were all generated by R software. Wilcoxon test was used to perform a difference comparison between the two groups. Machine learning was done with the ‘‘glmnet’’ and ‘‘BorutaR’’ packages in R. The CIBERSORT algorithm was used to complete the immuno-infiltration analysis. Correlation between key genes and immune cells calculated using the ‘‘Spearman’’ algorithm. *P*-values less than 0.05 were statistically significant.

## Results

### Shared DEGs were Identified

**Fig. 1:**
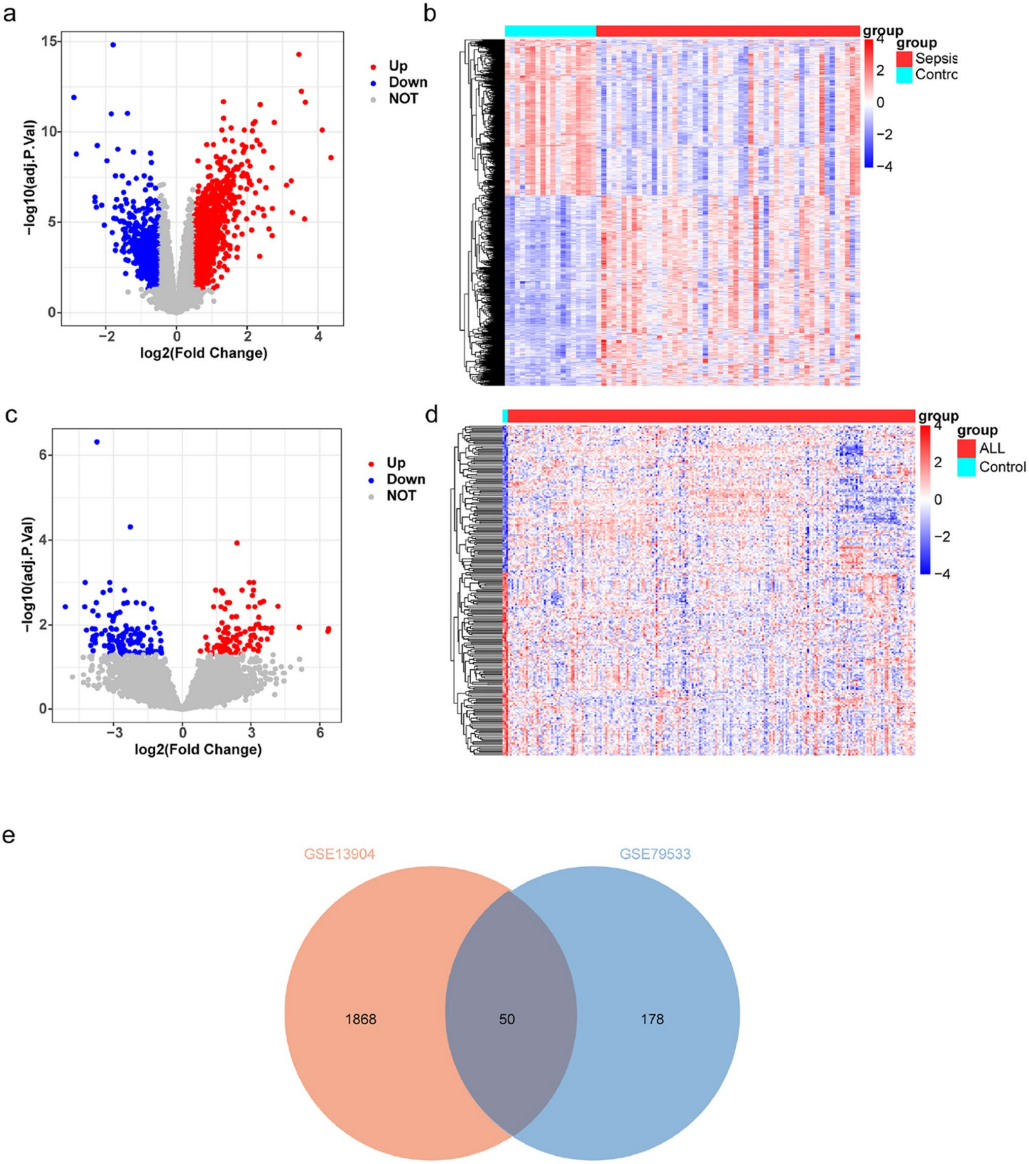
Collection of 50 shared differentially expressed genes (DEGs) common to GSE13904 and GSE79533. **a** Volcano map and **b** Heatmap of DEGs between patients with sepsis and control samples in GSE13904. **c** Volcano map and **d** Heatmap of DEGs between patients with ALL and control samples in GSE79533. **e** Venn diagram for the shared DEGs common to sepsis and ALL

A total of 1918 DEGs were obtained from GSE13904, of which 1055 DEGs were up and 863 DEGs down in sepsis (Fig. 1a–b). A total of 228 DEGs were obtained from GSE79533, of which 101 DEGs were up and 127 DEGs down in ALL (Fig. 1c–d). The 50 shared DEGs were obtained by taking the intersection of the DEGs obtained from GSE13904 and GSE79533 (Fig. 1e).

### Enrichment Analysis and Construction of the PPI Network

**Fig. 2:**
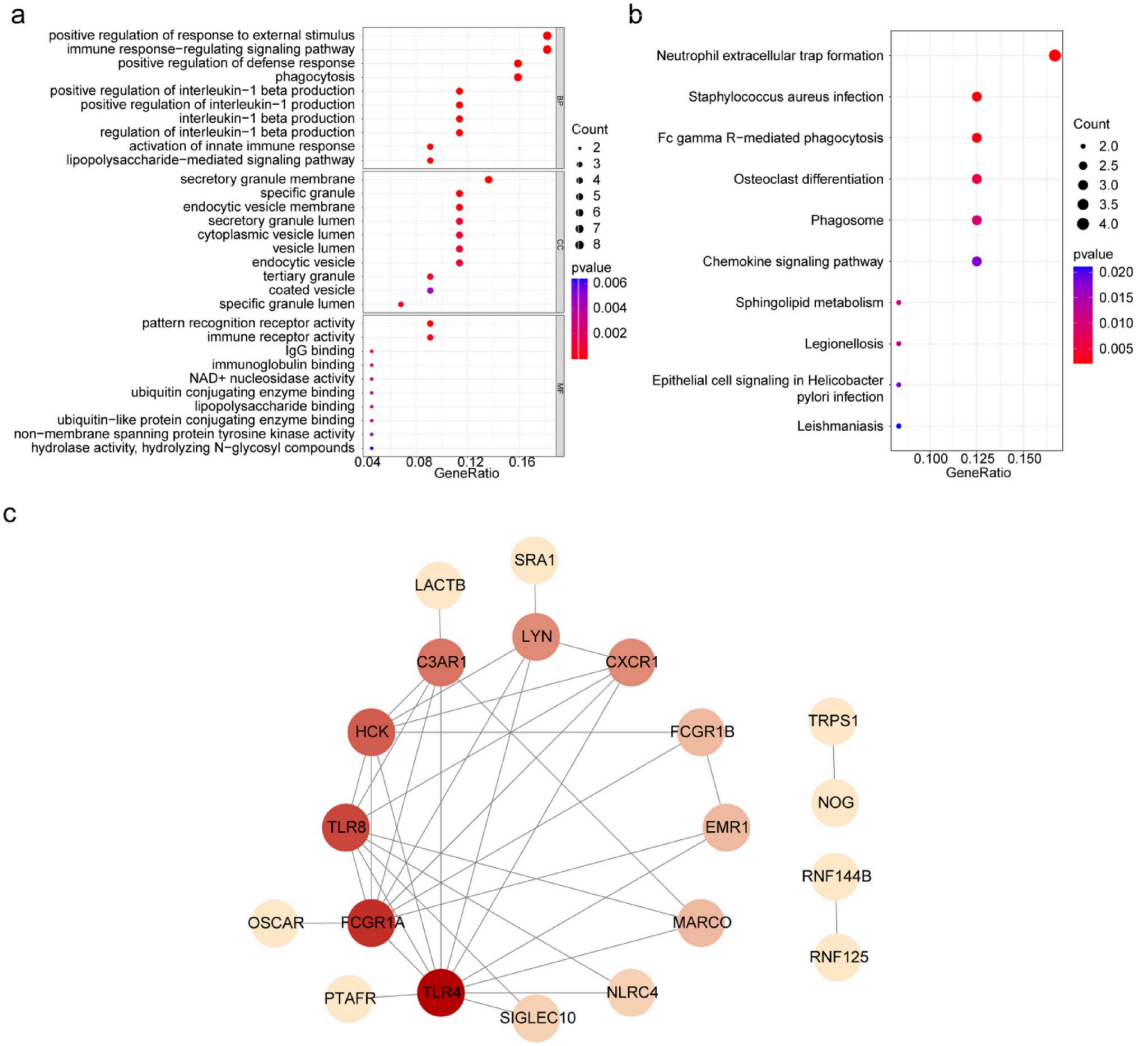
Exploring for the biological significance of 50 shared DEGs. **a** Gene Ontology (GO) and **b** Kyoto Encyclopedia of Genes and Genomes (KEGG) pathways analysis of shared DEGs. **c** Protein–protein interactions (PPI) network of shared DEGs

The GO entries involved in shared DEGs such as interleukin-1 beta production, and inflammatory response to wounding (Fig. 2a). The KEGG pathways involved in shared DEGs such as staphylococcus aureus infection, and th1 and neutrophil extracellular trap formation. This suggests that the shared DEGs may be affecting sepsis and ALL patients by influencing interleukin-1 beta production (Fig. 2b). To explore whether interactions existed among the shared DEGs, a PPI network was created with strong interactions between C3AR1 and LACTB, EMR1 and FCGR1B, and FCGR1A and TLR8 (Fig. 2c).

### HCK, NOG, RNF125 were Identified as Shared Key Genes

**Fig. 3:**
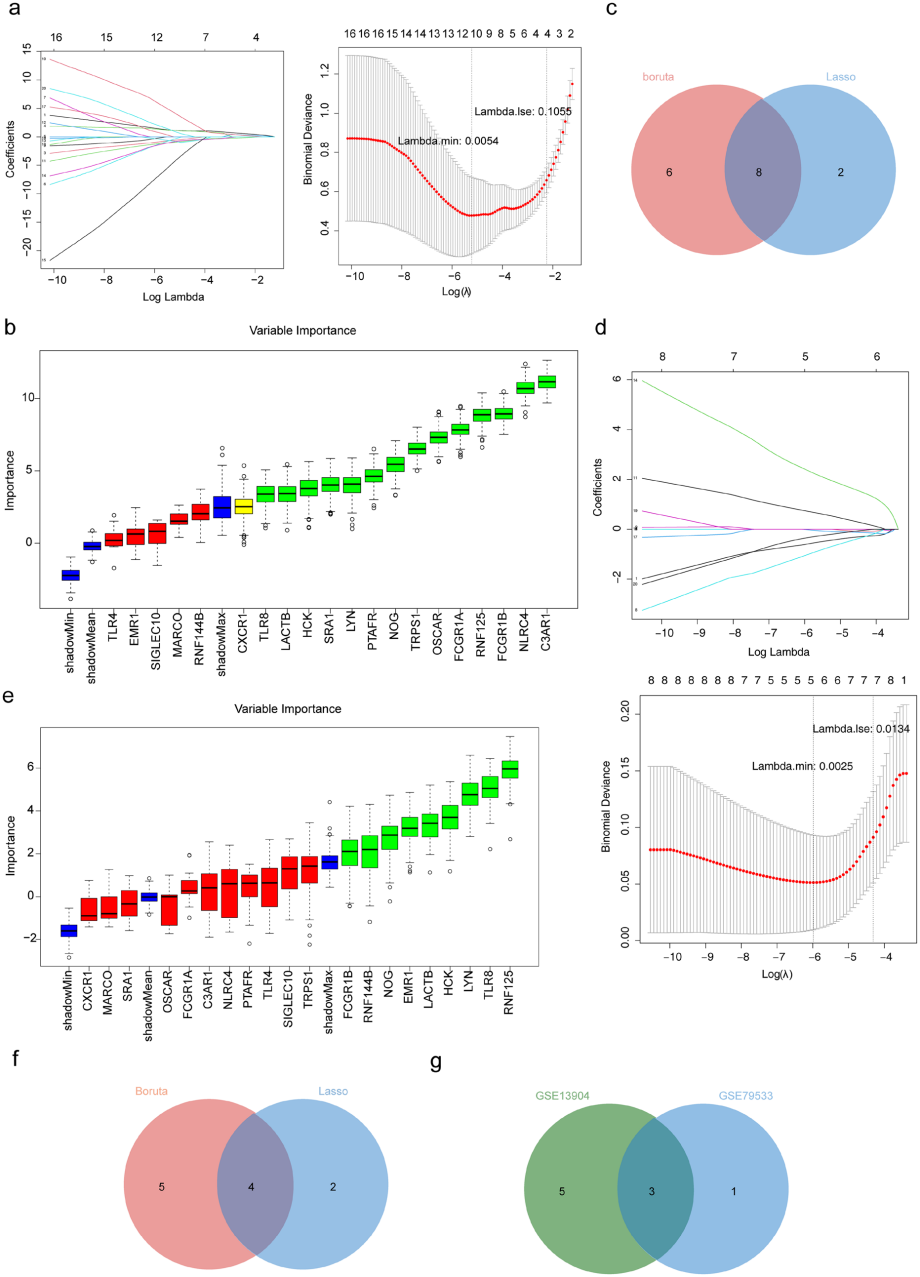
Analysis of shared key genes common to sepsis and ALL. **a** Least absolute shrinkage and selection operator (LASSO) regression analysis to screen ten characteristic genes in GSE13904. **b** Variable importance plot of the boruta analysis to obtain 14 signature genes. **c** Venn diagram for eight sepsis-related key genes common to two algorithms. **d** LASSO analysis captured six feature genes in GSE79533. **e** Variable importance plot of the boruta analysis to obtain nine characteristic genes. **f** Venn diagram for four ALL-related key genes common to two algorithms. **g** Venn diagram for three shared key genes common to sepsis and ALL

In the GSE13904 dataset, the results of the LASSO analysis were selected when lambda.min = 0.0054 and ten characteristic genes were obtained (Fig. 3a), and 14 characteristic genes were obtained by boruta algorithm (Fig. 3b). Eight sepsis-related key genes were obtained by taking the intersection of ten signature genes from LASSO analysis and 14 signature genes from boruta analysis (Fig. 3c). In the GSE79533 dataset, when lambda.min = 0.0025, the results of the LASSO analysis were selected and six feature genes were obtained (Fig. 3d). and nine characteristic genes were obtained by boruta algorithm (Fig. 3e). Four ALL-related key genes were obtained by taking the intersection of six signature genes from the LASSO analysis and nine from the Boruta analysis (Fig. 3f). Finally, three shared key genes were obtained by taking the intersection of eight sepsis-related key genes and four ALL-related key genes (Fig. 3g).

### Shared key Genes have Good Diagnostic Value in both Sepsis and ALL

**Fig. 4:**
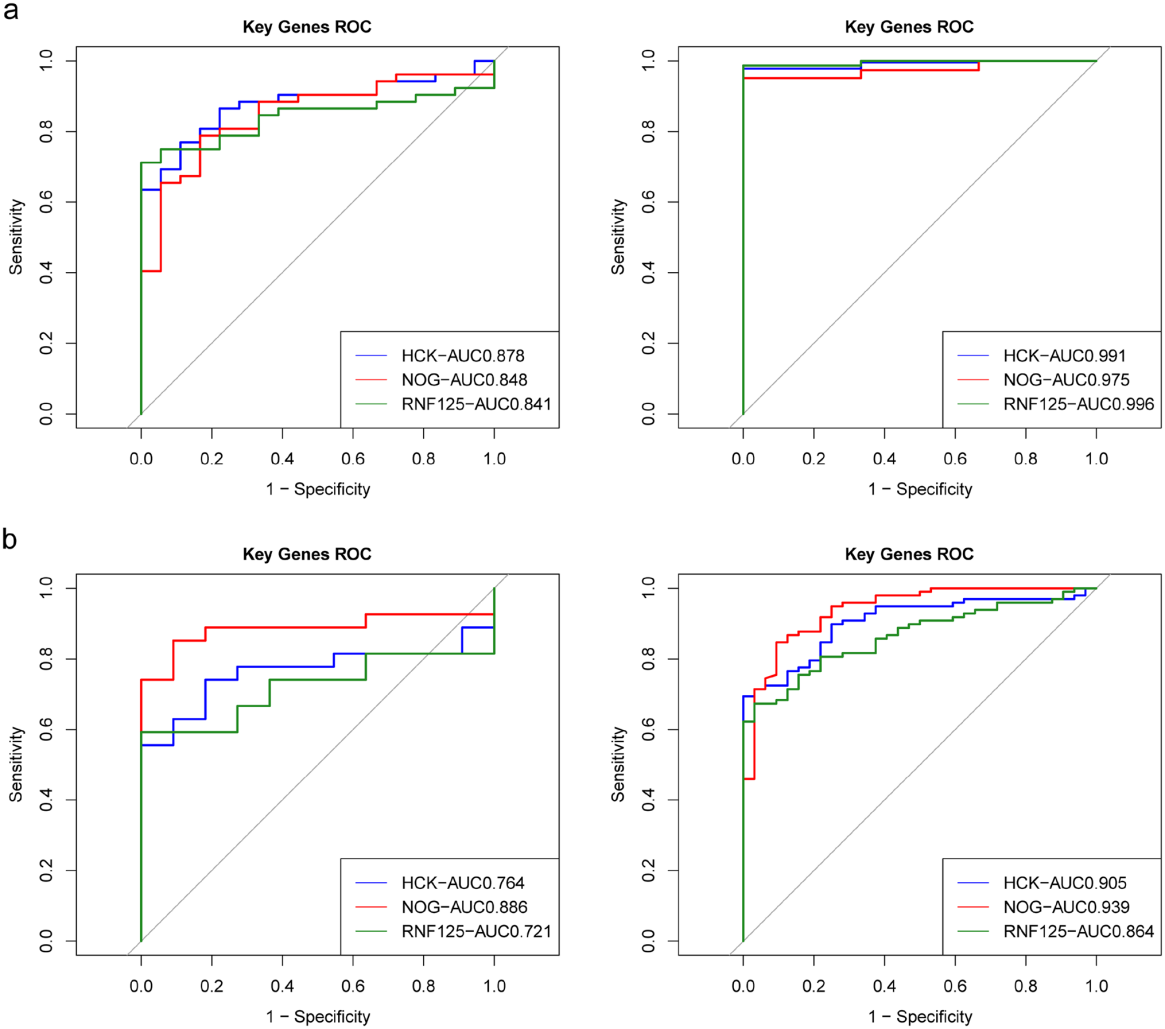
Receiver operating characteristic (ROC) curves of shared key genes in both sepsis and ALL. **a** ROC curves of three shared key genes in GSE13904 (sepsis) and GSE79533 (ALL). **b** ROC curves of three shared key genes in GSE26440 (sepsis) and GSE48558 (ALL)

The AUC of the ROC curves for the three shared key genes in the sepsis dataset GSE13904 and ALL dataset GSE79533 were all greater than 0.800 (Fig. 4a). Indicating that the shared key genes have a good diagnostic value for both sepsis and ALL, the ROC curves in GSE26440 and GSE48558 were validated this conclusion. The AUC of the ROC curves for all three shared key genes were greater than 0.800 in the sepsis dataset GSE26440 and greater than 0.700 in the ALL dataset GSE48558 (Fig. 4b).

### Different Relationships Between Shared key Genes and the Immune Environment

**Fig. 5:**
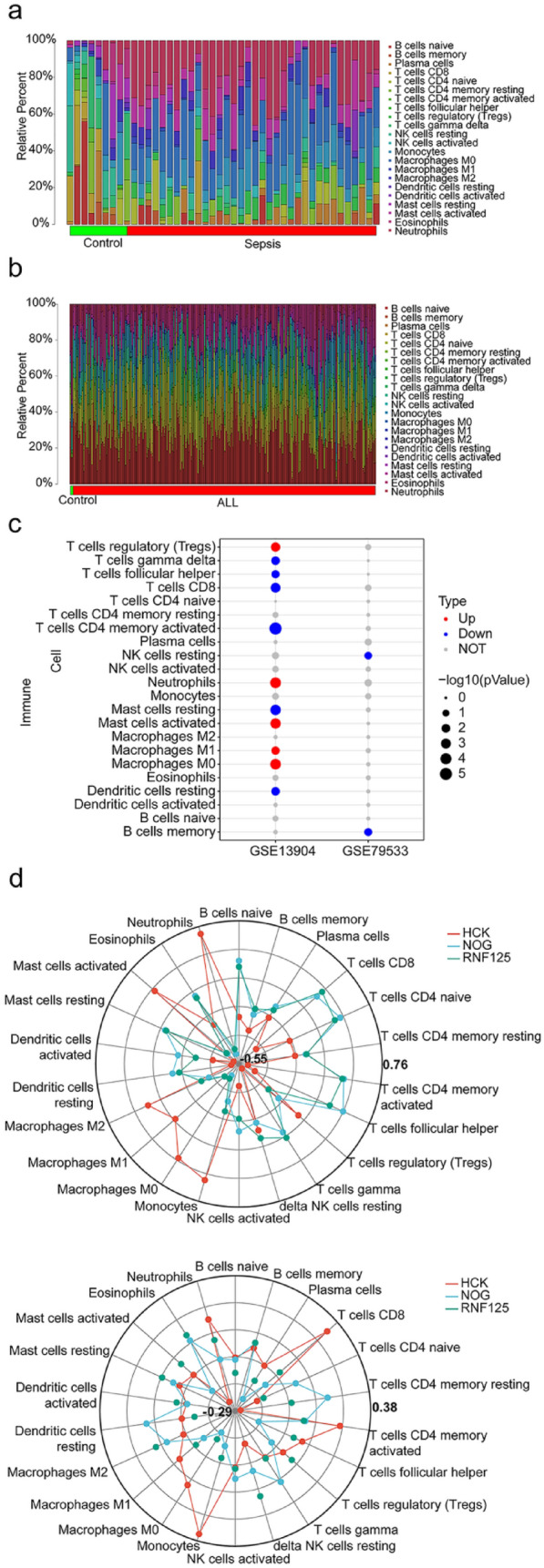
Differences in immune cell infiltration between sepsis and ALL. **a** Histogram for the immune cell proportions between control and sepsis groups. **b** Histogram for the immune cell proportions between control and ALL groups. **c** Bubble chart for differential expression of immune cells in GSE13904 and GSE79533. **d** Reda chart for the correlations between immune cells and shared key genes in GSE13904 and GSE79533

There were eleven immune cells that were different between sepsis and control samples in the GSE13904 (*P* < 0.05, Fig. 5a). There were two immune cells that were different between ALL and control samples in the GSE79533 (*P* < 0.05, Fig. 5b). The correlation between shared key genes and immune cells were higher in the septicemia dataset GSE13904 and conversely, the correlation between shared key genes and immune cells were lower in the ALL dataset GSE79533 (Fig. 5c–d).

### Expression Validation and Drugs Prediction

**Fig. 6:**
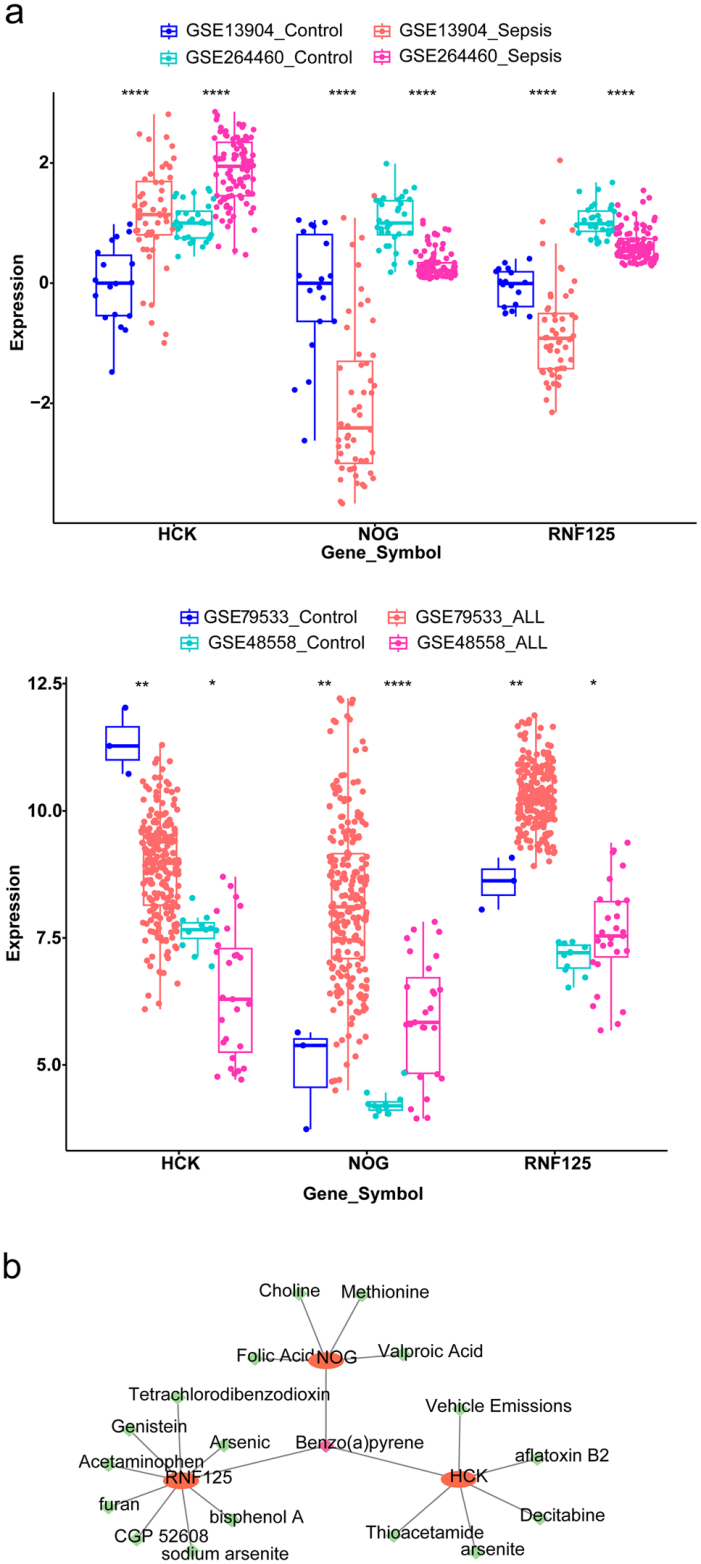
Expression patterns and drugs prediction of three shared key genes. **a** Box plots for differences of the gene expression in GSE13904, GSE26440 (sepsis), GSE79533, GSE48558 (ALL) datasets. **b** Gene-drug network targeting three shared key genes. *P < 0.05, **P < 0.01, ****P < 0.0001

The results of the expression validation showed identical expression trends for the three shared key genes in the sepsis datasets GSE13904 and GSE26440, and the ALL datasets GSE79533 and GSE48558 (Fig. 6a). The gene-drug network was shown in Fig. 6b, which included 18 drugs and 3 genes, and a total of 20 gene-drug pairs were obtained, such as HCK and aflatoxin B2, NOG and Benzo(a)pyrene.

## Discussion

Pediatric ALL is accompanied by low immunity, and the bone marrow suppression in the process of chemotherapy remarkably elevates the infection risk, which is likely to cause sepsis [12]. The sepsis in turn will exacerbate the development of pediatric ALL, hence a vicious cycle is created, bewildering clinicians in treatment. Nevertheless, rare literature has investigated the genetic association between sepsis and ALL, or the development of sepsis in ALL children. Hence, our study aims to explore the common genes as well as biological functions shared by sepsis and ALL, and deeply explain their molecular mechanisms, thereby addressing such practical problem facing clinical treatment.

HCK proto-oncogene (HCK), Src family tyrosine kinase, involves AIPCV, JTK9, p59Hck, p61Hck. The protein primarily shows hematopoiesis, especially in myeloid and B-lymphoid lineage cells. It is capable of coupling the Fc receptor to activated respiratory burst. According to Prateek Bhatia et al. [26], epigenetic analysis confirmed the hypomethylation and overexpression of protooncogenes-AXL, HCK, MED12, and ETS2 in relapsed cases of pediatric B-acute lymphoblastic leukemia. In the study by K Hoshino et al. [27], the promoter CpG island of Hck presents an aberrant methylation in leukemia, and patients who have Ph-negative ALL and Hck methylation present a weaker prognosis. All these confirmed the tumor-suppressive attributes of HCK in BCR-ABL negative leukemia. According to C A Lowell et al. [28], defective integrin signaling in neutrophils resulted from lost Hck and Fgr tyrosine kinase activity can cause inflammation-dependent tissue damage in vivo. On those account, HCK may significantly impact the pediatric ALL and sepsis.

Noggin gene [19] is also called as SYM1; SYNS1; SYNS1A. It takes charge of encoding secreted polypeptide for binding and inactivating the transforming growth factor-beta (TGF-beta) superfamily members, like the bone morphogenetic protein-4 (BMP4). Yongxing Lai et al. [29], after comprehensively analyzing the sepsis molecular subtypes and hub genes based on the gene expression profiles, confirmed NOG as a critical gene related to molecular subtypes of sepsis. According to previous studies, the change of NOG sequence impacts the mandibular development. In the study by Sandra J Gutiérrez-Prieto et al. [30], all the studies individuals lack methylation sites at the NOG gene promoter region, which demonstrates the possible regulatory effect of other epigenetic factors on the mandibular growth. Despite this, studies have not well explored the impact of NOG on childhood ALL, which shall be concerned in further studies.

Ring finger protein 125 (RNF125) includes TNORS, TRAC1 and TRAC-1, which takes charge of encoding a new E3 ubiquitin ligase to positively regulate the T-cell receptor signaling pathway. In the study by Juan Tang et al. [31], RNF125 is a kind of additional E3 ubiquitin ligase initiating NLRP3 LRR domain ubiquitination linked by K63, and the continuous NLRP3 ubiquitination by RNF125 and Cbl-b makes inflammasome less activated and restricts the endotoxemia. Despite the lack of studies reporting RNF125 and pediatric ALL, in the study by Takahiro Kodama et al. [32], RNF125 acts as a tumor suppressor with anti-proliferative attributes in hepatocellular carcinoma (HCC). On that account, RNF125 can positively help to inhibit ALL and pediatric sepsis.

Interleukin-1 beta (IL-1β) is a pleiotropic inflammation mediator generated under multiple stimuli. According to previous studies, the GABA transporter (GAT2) and serine metabolism assist in sustaining the production of IL-1β in macrophages [33, 34]. Also, based on the in vivo experiments, the deficient of GAT2 together with the pharmacological inhibition regarding de novo serine synthesis in vivo weaken the IL-1β level induced by LPS, and increase the sepsis survival rate in related LPS-driven model [33, 34]. Nevertheless, as revealed by E S de Bont et al. [35], the mean TNF-alpha and IL-1beta production per 1 × 10(4) monocytes do not present obvious differences in ALL children, or in controls. Also, considering the vital role played by IL-1beta in the antitumor immune response, it is believed that the common DEGs may together impact ALL and sepsis development among children by impacting the production of IL-1beta [36]. Tumor escape as well as other factors lead to weakened cellular immunity in pediatric ALL, and immune cell modification (CAR-T treatment, etc.) crucially impacts the refractory pediatric ALL [37]. Taken together, the common key genes possibly impact ALL and sepsis development among children through impacting immune cell expression.

The study is the first one that focuses on analyzing the crosstalk mechanism regarding ALL and pediatric septic blood at the transcriptome level, which involves autism spectrum disorder and inflammatory bowel disease [38], chronic kidney disease and ulcerative colitis [39]. Hence, it is rational to discuss the dual-disease joint, while experimental validation has not been provided. Our study gives a comprehensive analysis, involving the common gene signatures of HCK, NOG, and RNF125 in pediatric acute lymphoblastic leukaemia and pediatric sepsis, together with related biological mechanism. On that account, despite the absence of vivo and vitro validations, our study contributes to further relevant studies from theoretical perspectives.

The study mainly uses drugs of aflatoxin B2 and Benzo (a) pyrene (BaP), which, however, are slightly associated with the clinically commonly used drugs in ALL treatment for sepsis children. Aflatoxin B(2) is a kind of blue-fluorescent metabolite for Aspergillus flavus, isolated from the cultures cultured on the crushed wheat [40]. BaP has the function of regulating the immune microenvironment of lung, and can induce lung cancer [41]. It is necessary to further investigate the relationship between BaP, ALL and sepsis.

Despite the requirement for in-depth in vivo and vitro validations, the study results may assist future studies in investigating the association of pediatric ALL with sepsis theoretically.

## Conclusion

In summary, this study is the first to analyze pediatric ALL and pediatric sepsis through bioinformatics, and three shared key genes (RNF125, NOG, and HCK) have been obtained. Based on ROC curves of three shared key genes, we established a stable and accurate signature to evaluate the diagnosis of sepsis and ALL. Meanwhile based on gene-drug network analysis, RNF125, NOG and HCK can be used as potential therapeutic targets. In addition, we found that the correlation between shared key genes and immune cells were higher in the sepsis than ALL. This article contributes to a new perspective on the mechanisms involved in pediatric ALL and pediatric sepsis, but it has not been experimentally validated and we will also focus on this in future studies.

## Data Availability

The datasets used and analyzed in the current study are available from the GEO database (https://www.ncbi.nlm.nih.gov/gds) [GSE13904, GSE26440, GSE79533 and GSE48558].
